# Effectiveness of early interventions for parental sensitivity following preterm birth: a systematic review protocol

**DOI:** 10.1186/s13643-017-0459-x

**Published:** 2017-03-23

**Authors:** Andréane Lavallée, Marilyn Aita, Anne Bourbonnais, Gwenaëlle De Clifford-Faugère

**Affiliations:** 1 Faculty of Nursing, Université de Montréal, 2375 Chemin de la Côte-Sainte-Catherine, Montreal, Qc H3T 1A8 Canada; 2 Research Center of the CHU Sainte-Justine, Montreal, Canada; 3 Research Center of the Institut universitaire de gériatrie de Montréal, Montreal, Canada

**Keywords:** Infant, Premature, Parenting, Parent-child relations, Parental sensitivity, Interventions, Systematic review, Trials

## Abstract

**Background:**

Parental sensitivity is the interaction process by which parents (a) recognize cues from their infant, (b) interpret these cues adequately, (c) identify an appropriate response and (d) apply this response in an appropriate time frame. In the neonatal intensive care unit, parents of preterm infants often encounter factors hampering the establishment of their parental sensitivity. Parents report the need to be in proximity to and to participate in their preterm infant’s care in order to develop their sensitivity to their newborn infant. To do so, the effectiveness of interventions promoting their parental sensitivity has been evaluated with randomized controlled trials. The purpose of this systematic review is to evaluate the effectiveness of early interventions promoting parental sensitivity of preterm infants’ parents.

**Methods/design:**

A search will be done in the following databases: CINAHL, PubMed in addition to Medline, Embase, PsycInfo, Web of Science, Scopus and ProQuest. No restriction for the years of publication will be considered. Two experts will be conducting independently each step of the review. All studies of randomized controlled trials of early interventions, for parents of preterm infants, implemented in the neonatal intensive care unit before the infant has reached 37 weeks of corrected gestational age, will be considered eligible. Primary outcome is parental sensitivity. Depending on the availability and quality of data, a meta-analysis will be done. Alternatively, a qualitative synthesis of data is planned. The systematic review follows the PRISMA recommendations. Finally, risk of bias and quality of the evidence of included studies will be assessed.

**Discussion:**

To our knowledge, this will be the first systematic review to examine the effect of early interventions that promote parental sensitivity of parents of preterm infants in the neonatal intensive care unit. The results of this review will guide development of best practice guidelines and recommendations for further research and will have implications for neonatal clinical practice.

**Systematic review registration:**

PROSPERO CRD42016047083

**Electronic supplementary material:**

The online version of this article (doi:10.1186/s13643-017-0459-x) contains supplementary material, which is available to authorized users.

## Background

Parental sensitivity is the interaction process by which parents (a) recognize cues of their infant, (b) interpret these cues adequately, (c) identify an appropriate response and (d) apply this response in an appropriate time frame [1]. Parental sensitivity predicts long-term attachment [2] which needs to develop on a long-term basis [3]. Parental attachment also plays a role in enhancing long-term neurological development in infants [4, 5]. However, it is well known that parental sensitivity can be compromised in the neonatal intensive care unit (NICU) for several reasons, such as the high amount of stress parents have to go through during their preterm infant’s hospitalization [2] and the loss of their parental role [6]. These factors result in constraining parents from participating in their preterm infant’s care and contribute to their loss of parental confidence [7]. Parents of preterm infants hospitalized in the NICU often report a need to have physical contacts with their infant and a desire to participate in their infant’s care [8]. Such parents also report the need for support to be able to do so [8]. Therefore, interventions to promote parental sensitivity during NICU hospitalization, as well as enhance neurological development in these infants, have been developed and evaluated in experimental or quasi-experimental studies. In fact, a systematic review and meta-analysis of 25 randomized controlled trials (RCT) examining the effect of early interventions aimed at enhancing parental involvement in their preterm infant’s care show an overall positive effect on the preterm infant’s neurological development lasting up to 36 months of corrected age [9]. More specifically, a systematic review and meta-analysis were conducted in 2003 examining the effect of early interventions to promote parental sensitivity in parents of preterm and term infants [3] and show that a wide range of early parental interventions promoting parental sensitivity can be effective in enhancing long-term attachment [3]. For example, interventions using video feedback appear to be more effective than any other types of interventions [3]. A major drawback has been that term and preterm infants were not separated in the meta-analysis and studies included in the review were mainly conducted with a term infant population. As a result, the conclusions drawn from this meta-analysis [3] are not generalizable to guide the development of interventions to promote the use of parental sensitivity programmes for parents following a preterm birth. Benzies and colleagues [10] also published a systematic review and meta-analysis looking at effects of early interventions for preterm infants involving parents, where sensitivity has been reported as a parental outcome. The meta-analysis including six studies shows no significant results favouring the interventions. Although these results are of interest, studies included in the meta-analysis do not extensively report the components of the interventions. Also, more RCTs focused on these types of interventions reporting parental sensitivity as an outcome have been published and are not included in the meta-analysis. Results of such a systematic review including more studies evaluating interventions promoting parental sensitivity following preterm birth would guide the neonatal clinical practice and research to promote parental sensitivity and, hence, parent-infant attachment and neurological development of preterm infants. Therefore, the aim of this systematic review is to evaluate the effectiveness of early interventions promoting parental sensitivity of parents of preterm infants during NICU hospitalization.

### Objective

The objective of the systematic review will be to answer the following question: What is the effectiveness of early interventions for parents of preterm infants on parental sensitivity, compared to standard care?

## Methods

To enhance transparency, this systematic review protocol has been developed according to the Preferred Reporting Items for Systematic review and Meta-Analysis Protocols (PRISMA-P) [11] [see Additional file 1]. This protocol has also been registered on the PROSPERO database (registration number: CRD42016047083).

### Eligibility criteria

Primary studies published in French or English will be included in this systematic review if they meet the following criteria.

#### Type of studies

Only RCTs will be included in the systematic review. No limit regarding the years of publication will be considered because this will be the first systematic review looking at early interventions for parental sensitivity following preterm birth during NICU hospitalization.

#### Participants

Primary studies eligible for this review will have included the following participants: infants born at 36^6/7^ weeks of gestation or less, their mothers, fathers or both parents.

#### Interventions

The included primary studies will consist of interventions started in the NICU, before the preterm infant has reached 37 weeks corrected age. Interventions will necessarily be done with the mother, the father or both parents of preterm infants. Parents will have had an active role or a passive role, as they will have either participated in the intervention (active) or received educational content (passive). More specifically, when parents will be considered active, they will for example be participating in their preterm infant’s care or interacting with him/her. When the parents will be considered passive, they will be receiving, for instance, information on preterm infants’ cues, care, etc. In the latter, health care professionals will be actively involved in the intervention by providing information. No limitation according to the dose, length and follow-up of the intervention will be considered. As previously cited, only early interventions which will have started before the neonatal intensive care unit discharge will be considered eligible. All types of interventions will be considered eligible to be included in the review. For example, early sensitivity training programmes such as interventions where parents learn to read their infant’s cues, participate in their care or learn to interact with them will be included. If the intervention is not adequately described, authors will be contacted.

#### Comparator

All types of comparator groups will be included in this systematic review whether they are a non-exposed control group or a group exposed to another intervention if applicable.

#### Primary outcome

Studies measuring parental sensitivity as a primary outcome will be included. For this review, parental sensitivity is defined as (a) identifying cues of their preterm infant, (b) interpreting the cue adequately, (c) identifying an appropriate response and (d) applying this response in an appropriate time frame [1]. Noteworthy, the term “sensitivity” is often used interchangeably in the literature with other terms such as parental attachment. Only studies reporting parental sensitivity as previously defined will be included in this review. All types of standardized tools to measure parental sensitivity will be considered whether they are self-reported tools or observational measures of parental sensitivity behaviours (verbal and/or non-verbal). When observational measures are used, behaviours are observed and scored live or videotaped. When videotaped, the behaviours are scored later. In both live and videotaped scoring, validated scales are used (Nursing Child Assessment Teaching Scale, Nursing Child Assessment Feeding Scale, Care Index, NICHD scale or Global rating scale). Parental sensitivity will be measured at time of discharge or as a long-term follow-up post-discharge which will be considered as a subgroup analysis.

#### Secondary outcome

Neurodevelopment will be considered as a secondary outcome if it was measured in the primary study where parental sensitivity was also measured. Primary studies will not be included in the review if neurodevelopment was measured but not parental sensitivity. All types of standardized instruments, scales or tests used to measure neurodevelopment will be considered. Neurodevelopment will be considered as a neurobehavioral measure (using Neonatal Behavioural Assessment Scale or Neurobehavioural Assessment of the Preterm Infant) or measured by a magnetic resonance imaging (MRI).

### Information sources

The literature search will be done in a wide range of electronic databases to ensure complete coverage: CINAHL, PubMed and Medline, Embase, PsycInfo, Web of Science, Scopus and ProQuest. We will also look at the website <clinicaltrials.gov> to look for relevant RCTs registered and not yet completed or published. To decrease the risk of publication bias [12, 13], additional research will be done in relevant reference lists. Also, journals and articles published by authors known to us as working on interventions for promoting parental sensitivity will be examined.

### Search methods

The search strategy was developed in collaboration with a nursing and allied health sciences librarian with experience in systematic reviews. The search strategy includes MESH Terms and keywords, i.e. parental sensitivity, preterm infant and their synonyms [see additional file 2]. The electronic search was limited to English and French literature.

### Study records

Data management will be done using EndNote©. The selection process will be conducted by two experts independently, after duplicates will have been deleted by the first author. Steps proposed by Pai and his colleagues [14] will be followed. A first selection of relevant studies will be done independently according to the titles and abstracts. Disagreements will be resolved by consensus, and a third expert will be involved in case where a disagreement cannot be resolved by the two first experts. After the first screening is complete, the full text of eligible articles will be gathered through online institutional access to articles from various databases. Authors of unavailable articles will be contacted to obtain access to the article of interest. Selected articles, after the first screening, will then be screened for a second time independently by the two experts according to the full article. Excluded articles, after the second screening, will be documented with reasons for exclusion. Again, disagreements will be resolved by consensus. If consensus cannot be reached, a third expert will be involved to solve disagreement. Articles included after the full-text review will be given a unique ID number before data will be extracted. The PRISMA flow diagram will be used to report the selection process.

### Data extraction and management

A paper report form will be developed by the two experts for data extraction. Extracted data then will be put into the Review Manager software (RevMan 5.1). Data will be extracted independently by the two experts. Authors of the included primary studies will be contacted by email for missing data. If missing data cannot be obtained, they will be entered with replacement values [15].

Extracted data will include

- Information on the published article: year of publication, country of publication, journal and authors
- Information on the study: study design, sample size, attrition rate, number of centres included, limits, tools used to measure primary outcome and outcome measures
- Information on the intervention: name of the intervention, programme components, number of sessions, duration of each session, duration of the whole programme, status of the intervention provider, control group components, specific population (gestational age (GA) at involvement in the study, GA when the intervention occurred, which parent included) and theoretical framework
- Results according to our primary (parental sensitivity) and secondary outcomes (preterm infant’s neurological development)

### Risk of bias assessment

Risk of bias will be assessed for each primary study included in the systematic review using the Cochrane Collaboration Risk of Bias Assessment Tool [15, 16]. The tool consists of seven sources of bias, which each will be scored as low, high or unclear risk of bias. The eight sources of bias are random sequence allocation, allocation concealment, blinding of participants and personnel, blinding of outcome assessment, incomplete outcome data (patient-reported outcomes and all-cause mortality), incomplete outcome data and selective reporting. Results of this assessment will be reported in a table included in the systematic review. The risk of bias assessment will be done by the two experts independently. Again, in case of disagreement among the two experts, consensus will be used to solve the disagreement.

### Data synthesis

Data will initially be qualitatively synthesized. The results of the RCTs will therefore be presented with a qualitative description. If at least two studies are available, a meta-analysis will be conducted. Continuous variables will be analysed using weighted mean differences with a 95% confidence interval. Categorical variables will be analysed using counts and percentages. Using the RevMan software, a fixed-effects model will first be used for meta-analysis. Heterogeneity will be assessed with the *I* ^2^ statistic within RevMan. The *I* ^2^ statistic will be interpreted as the following: 0 to 40% heterogeneity might not be important, 30 to 60% may represent moderate heterogeneity, 50 to 90% may represent substantial heterogeneity and 75 to 100% considerable heterogeneity [17]. Interpretation of the *I* ^2^ statistic will be done by the two experts by consensus. The chi-squared test (*x* ^2^) will also be used to assess heterogeneity. A *p* value of 0.10 or smaller will be considered to determine statistical significance [15]. If the *p* value >0.10, if heterogeneity is interpreted as being not important or if moderate heterogeneity (*I* ^2^ < 50%) can be explained, a random-effects model will be used for meta-analysis. A sensitivity analysis will be performed to try to explain heterogeneity depending on the interpretation of the *I* ^2^ and *x* ^2^ values. If heterogeneity cannot be explained by the sensitivity analysis, the fixed-effects model will be used for meta-analysis [15]. Additional analysis will be conducted such as subgroup analysis based on age of preterm infants at time of data collection. A funnel plot and the statistical test of Egger will also be performed to assess for publication bias. If heterogeneity is too important and cannot be explained, thus making meta-analysis impossible, data will only be qualitatively synthesized.

### Quality of evidence

As suggested by the PRIMA-P group [17], the quality of each included trial will be determined using the GRADE system [18, 19]. The GRADE system will also allow us to rate the strength of the evidence [18]. The two experts will rate the quality as well as the strength of the evidence using the GRADE system independently and disagreements will be solved by consensus.

## Discussion

The primary objective of this systematic review and meta-analysis is to review the interventions to promote parental sensitivity, following preterm birth, during NICU hospitalization. Strengths and weaknesses of the review will be discussed and recommendations for research and/or for clinical practice will be provided in order to promote parental sensitivity in this specific context.

## Additional files

Additional file 1: PRISMA-P 2015 checklist. (DOCX 34 kb)

Additional file 2: CINAHL search strategy. (DOCX 130 kb)

## Abbreviations

PRISMA-P: Preferred Reporting Items for Systematic review and Meta-Analysis Protocols
NICU: Neonatal intensive care unit
RCT: Randomized controlled trial
