# Supplementary material for: Effectiveness of early interventions for parental sensitivity following preterm birth: a systematic review protocol
Source: Syst Rev. 2017 Mar 23;6:62. doi: 10.1186/s13643-017-0459-x (PMC5364600; doi:10.1186/s13643-017-0459-x)
Supplement: Supplementary file 2 — CINAHL search strategy. (DOCX 130 kb) [file 13643_2017_459_MOESM2_ESM.docx]

**Additional file 2**

**Table1**

**CINAHL search strategy**

| **Themes** | **Parental sensitivity** | **Preterm infants** |
| --- | --- | --- |
| ***Subject terms* and key words** | S1: (MH "Attachment Behavior") OR (MH "Parent-Infant Bonding")  S2: (MH "Parent-Infant Relations") OR (MH "Father-Infant Relations") OR (MH "Mother-Infant Relations") OR (MH "Family Relations")  S3: (MH "Patient-Family Relations") OR (MH "Nurturing Behavior")  S4: (MH "Paternal Behavior") OR (MH "Parenting")  S5: (MH "Maternal Behavior")  S6: (MH "Family Attitudes") OR (MH "Parental Attitudes") OR (MH "Maternal Attitudes") OR (MH "Paternal Attitudes")  S7: (MH "Parent-Child Relations") OR (MH "Father-Child Relations") OR (MH "Mother-Child Relations")  S9: (MH "Parenting")  S10: S1 OR S2 OR S3 OR S4 OR S5 OR S6 OR S7 OR S9 (total 59 915 articles)  S8: TI ( (((parent* OR maternal OR paternal OR mother* OR father*) N4 (attach* OR sensitiv* OR responsiv* OR interact* OR behav* OR bond* OR intrusiveness OR social* OR relation*))) ) OR AB ( (((parent* OR maternal OR paternal OR mother* OR father*) N4 (attach* OR sensitiv* OR responsiv* OR interact* OR behav* OR bond* OR intrusiveness OR social* OR relation*))) ) OR MW ( (((parent* OR maternal OR paternal OR mother* OR father*) N4 (attach* OR sensitiv* OR responsiv* OR interact* OR behav* OR bond* OR intrusiveness OR social* OR relation*))) ) (39 570 articles) | S12: (MH "Neonatal Nursing") OR (MH "Neonatal Intensive Care Nursing")  S13: (MH "Intensive Care, Neonatal") OR (MH "Kangaroo Care")  S14: (MH "Intensive Care Units, Neonatal")  S15: (MH "Infant, Low Birth Weight") OR (MH "Infant, Small for Gestational Age") OR (MH "Infant, Very Low Birth Weight") OR (MH "Infant, Premature")  S16: (MH "Infant, High Risk") OR (MH "Infant, Hospitalized")  S17: (MH "Neonatal Assessment") OR (MH "Brazelton Neonatal Behavioral Assessment Scale")  S18: (MH "Childbirth, Premature") OR (MH "Labor, Premature")  S19: S12 OR S13 OR S14 OR S15 OR S16 OR S17 OR S18 (41 209 articles)  S20: TI ( (neonat* OR ((prematur* OR preterm OR "pre-term" OR (low* N2 weight) OR hospitalized OR "high risk" OR small) n4 (newborn* OR infant* OR baby OR babies))) ) OR AB ( (neonat* OR ((prematur* OR preterm OR "pre-term" OR (low* N2 weight) OR hospitalized OR "high risk" OR small) n4 (newborn* OR infant* OR baby OR babies))) ) OR MW ( (neonat* OR ((prematur* OR preterm OR "pre-term" OR (low* N2 weight) OR hospitalized OR "high risk" OR small) n4 (newborn* OR infant* OR baby OR babies))) ) (64 897 articles). |
| **AND / OR (results)** | S11: S8 OR S10 (71 133) | S21: S19 OR S20 (70 013) |
| **Final search strategy (results)** | S22: S11 AND S21 (3 647) | |
